# Improving the odds together: a framework for breast cancer research scientists to include patient advocates in their research

**DOI:** 10.1038/s41523-022-00440-y

**Published:** 2022-06-30

**Authors:** Hillary Stires, Igor Bado, Thelma Brown, Martha Carlson, Isaac S. Chan, Gloria V. Echeverria, Andrew J. Ewald, Bora Lim, Carla Lloyd, Julia Maues, Steffi Oesterreich, Robert N. Riter, Kelly Shanahan, Alana L. Welm, Josh Newby

**Affiliations:** 1 Friends of Cancer Research, Washington, DC USA; 2 Baylor College of Medicine, Houston, TX USA; 3 Unaffiliated Patient Advocate, Birmingham, AL USA; 4 Unaffiliated Patient Advocate, Chicago, IL USA; 5 University of Texas Southwestern, Dallas, TX USA; 6 Johns Hopkins University School of Medicine, Baltimore, MD USA; 7 Unaffiliated Patient Advocate, Salt Lake City, UT USA; 8 GRASP, Washington, DC USA; 9 University of Pittsburgh, Pittsburgh, PA USA; 10 Unaffiliated Patient Advocate, Ithaca, NY USA; 11 METAvivor, Lake Tahoe, CA USA; 12 University of Utah Huntsman Cancer Institute, Salt Lake City, UT USA; 13 Theresa’s Research Foundation, Houston, TX USA

**Keywords:** Metastasis, Preclinical research, Breast cancer

## Abstract

Including patient advocates in basic cancer research ensures that breast cancer research is intentional, supports effective communication with broader audiences, and directly connects researchers with those who they are striving to help. Despite this utility, many cancer research scientists do not work with patient advocates. To understand barriers to engagement and build a framework for enhanced interactions in the future, we hosted a workshop with patient advocates and researchers who do engage, then discussed findings at an international metastatic breast cancer conference to solicit additional feedback and suggestions. Findings demonstrate that researchers are uncertain about how to initiate and maintain relationships with advocates. We offer actionable steps to support researchers working with patient advocates to improve cancer research and accomplish our collective goal of improving lives of those who have been diagnosed with breast cancer. We hope that this initiative will facilitate such collaborative efforts.

## Introduction

Teams with diverse perspectives drive success when finding effective and efficient solutions for the biggest problems in healthcare^1,2^. Patients living with cancer advocate for treatments and research that are impactful and patient-centered^3^. Cancer research requires multidisciplinary teams, and biologists, biostatisticians, and clinicians are often collaborators on both preclinical and translational projects. It is a natural corollary that cancer scientists should incorporate the patient voice in their research to ensure the downstream impacts of their work serve those whose lives their research strives to improve^4–6^.

There are a variety of initiatives that describe the value of patient advocates and research scientists working together, some of which provide patient advocates with resources for establishing relationships with researchers^3,7–10^. However, there is a lack of guidelines or resources for how to initiate working with patient advocates to build successful long-term relationships. As such, the focus of our paper provides a framework that encourages research scientists to work with patient advocates to expedite and prioritize cancer research that improves patient outcomes.

Throughout this document we use the term “patient advocate” to describe people who have been diagnosed with cancer (or their caregivers) and work with research scientists. As a best practice, researchers should ask patient advocates they work with about their preferred terminology. For the purposes of our discussion, “research scientists” or “researchers” are those whose work focuses on cancer and can span multiple disciplines from biophysics to chemistry to cell biology to clinical trials.

## Results

### Encouraging researchers to work with advocates

Overall, we found that successful partnerships help focus research on meaningful outcomes, educate laboratory-based researchers about clinical care, provide motivation for effort, and aid dissemination of results. Patient advocates bring their personal experience of living with the disease and have dedicated time and energy to better understand cancer research. They seek to work with researchers to improve the outcomes for people living with cancer and to both speak on behalf of themselves and as representatives of a larger community. The goal of these collaborations is to help researchers improve as individuals through improved communication skills and by working directly with those they hope their research helps. Additionally, listening to patient advocates impacts cancer research by ensuring it is patient centric and focused on the needs of the community. There are several training programs for patient advocates to learn how to work with research scientists; however, the community would benefit from additional training for advocates that can be accessed in a more equitable way (e.g., low/no cost, virtual meetings, easy to find and enroll, publicly available).

### Value of researchers working with advocates

As experts in their experience with their disease, patient advocates are knowledgeable about the current treatment landscape and can help researchers understand what happens in the clinic. Research scientists who work with patient advocates find the partnership helps them to focus their work on the translational objective and keeps them grounded in thinking about its direct impact on patients, thereby creating a sense of urgency. Science communication and science literacy are increasingly important as information becomes more publicly accessible. Thus, working with patient advocates provides research scientists with an opportunity to practice communicating with people other than scientists.

### Considerations for contextualization

The group involved in the workshop was largely comprised of researchers and patient advocates with a focus on metastatic breast cancer in the United States. There is an increasing emphasis on metastasis research because the majority of cancer mortality is due to metastasis^11^. While most cancer research does not specifically focus on metastatic disease, lessons learned from research on metastatic disease broadly impact our understanding of cancer biology. Metastatic breast cancer patient advocates provided their perspectives to lay the groundwork, however, we appreciate that the findings from this group may not be reflective of other researchers and patient advocates in other cancer settings. We anticipate that our findings can be extrapolated to other diseases and believe that more research in other settings will be valuable to the field.

## Discussion

We discussed challenges to initiate and maintain researcher–advocate relationships, and proposed short- and long-term solutions (Table 1):

**Table 1:** Challenges and opportunities for establishing relationships between advocates and researchers.

Barrier	Short-term goal	Long-term goal
It is not always clear why patient advocates should be included in research	Encourage granting agencies to require including patient advocates in grant applications and provide a statement about the value of these partnerships such as the American Cancer Society (ACS), the DOD, and Susan G. Komen	Perform a quantitative research project to demonstrate the value of patient advocates and researchers working together
Researchers are worried about saying the wrong thing	Support spaces where open communication is encouraged	Create training programs for research scientists to learn how to work with patient advocates and communicate effectively
Researchers do not know how to begin working with patient advocates	Connect with patient advocates through Twitter, consider following social media chats such as #BCSM, #LCSM, and others; Attend conferences that patient advocates attend and engage through programs like GRASP	Develop a platform that would connect researchers and patient advocates nationwide
Researchers do not know how to include patient advocates in research	- Review existing programs from peer institutions that support patient advocate inclusion in research to determine the potential value at your institution; ﻿Consider activities that support longstanding relationships within the time commitment both parties are available such as journal clubs, practicing elevator pitches, writing lay abstracts, and inviting patient advocates to laboratory meetings	Request that groups who require patient advocate involvement provide compensation rubrics; Ask that NCI update grant and comprehensive cancer center designation rubrics to incorporate working with patient advocates

The first challenge is that it is not clear why patient advocates should be included in research. Sometimes researchers feel that their work is too far removed from the patient or that the patient advocate may not be able to provide anything valuable to the research. There are plenty of resources that describe the value of these relationships, but more can be done to ensure the value is demonstrated^8,12,13^.

In the short-term, anecdotal stories can support researchers’ realization of the value of working with patient advocates. Groups that require patient advocate involvement for funding, such as the Department of Defense Breast Cancer Research Program, should supply clear, public statements describing the benefit of including patient advocates in the research process. All disease-related research funding groups should encourage or require patient advocate input and involvement in grant projects, both to support writing the project plan and as part of developing the hypothesis, designing the project, and supporting dissemination of the findings. Researchers should ensure that they avoid engaging in “performative advocacy” (having a “token advocate”) through clear and frequent communication with the patient advocate. Performative advocacy is when patient advocates are included in research discussions to fulfill a requirement by simply saying a patient advocate was present, rather than listening to the patient advocate’s suggestions and incorporating their feedback into the study.

In the long-term, it would help to initiate a research project whose goal is to evaluate the added benefit of researchers working with patient advocates. This type of work should consider endpoints such as funding, publications, and overall satisfaction with work. Additionally, clinical endpoints may also improve and thus should be measured including translation to the clinic and engagement of diverse populations in related trials.

The second challenge is that researchers are worried about saying the wrong thing. During the panel discussions, one researcher said, “it’s intimidating. I don’t want to fail the patients. I want what I’m doing to be meaningful.” Some researchers fear working with a patient advocate in case they want a cure or have unrealistic expectations of how quickly (or how slowly) the research is going to move, which may feed into the researcher’s concern of failure. Metastatic disease is currently not curable, and many (but not all) patients will have limited time to live, and thus many researchers fear that they will say something that is insensitive given this challenging situation.

Open lines of communication are extremely important as they establish expectations from the start that everyone is learning from each other and ensure the environment is a safe space. Both groups should feel that they can ask questions they may feel like others know the answer to. They should also feel comfortable respectfully correcting others within their own areas of expertise. Our discussions noted that scientists have not historically been trained to communicate with nonscientists, but there is increasing recognition of this shortcoming, and current trainees will hopefully reap the benefits of increased attention to this matter^13,14^.

There is a need for training programs in which researchers learn how to work with patient advocates. This could be a workshop at a conference or annual retreat, or part of a class about communication. Conferences like the American Association for Cancer Research (AACR) Annual Meeting, the American Society for Clinical Oncology (ASCO) Annual Meeting, the San Antonio Breast Cancer Symposium (SABCS), and the Metastasis Research Society (MRS) Biennial Congress should consider including training sessions on best practices for working with patient advocates at their events.

The third challenge is that researchers do not know where to meet patient advocates. Initially, connecting with patient advocates can be challenging for researchers. It can happen through a variety of venues, though, through formal programs like those at Georgetown University^8^ and Huntsman Cancer Institute or informally through forums like Twitter by following #BCSM and other cancer-specific social media tags.

In the short-term, researchers should review established advocate programs to determine if they would benefit from initiating similar programs at their institutions. Georgetown University and Huntsman programs are examples where patient advocates meet regularly to support research at their respective cancer centers by providing a forum for researchers to explain their work and meet new patient advocates. Patient advocates could be invited to annual retreats to give those who do not normally work with advocates a chance to establish collaborations. Kansas University developed a researcher/patient advocate toolkit called PIVOT (Patient and Investigator Voices Organizing Together) Advocate↔Researcher Working Together Toolkit^15^, which provides additional best practices for establishing relationships. The National Cancer Institute Specialized Program of Research Excellence (SPORE) grants are structured to bring basic research to a Phase I Trial and SPORE applications are required to include advocates.

There are scientific conferences that support patient advocate participation and interaction with researchers such as the Metastatic Breast Cancer Research Conference and AACR’s Scientist↔Survivor Program. Additionally, an organization run by patient advocates called Guiding Researchers and Advocates to Scientific Partnerships (GRASP) fosters patient advocate and researcher interactions through poster discussions at conferences. Conferences also benefit from facilitating opportunities for patient advocates and research scientists to have formal interactions through panel discussions and informal interactions like coffee chats or happy hours.

In the long-term, it would be beneficial to develop a national database for researchers and patient advocates to connect that includes the person’s interests, location, and time commitment. Groups like GRASP have already started this type of database that includes hundreds of patient advocates and researchers. It would be helpful if this database were turned into a tool or phone app that connects researchers and patient advocates through a series of questions similar to something like match.com. Developers should work towards being inclusive of research on multiple cancer types and consider piloting the tool or app in a specific region to optimize it before opening it up nationwide.

The final challenge is that researchers do not know how to include patient advocates in research. Once a researcher has met a patient advocate, they may not know how to continue to include the patient voice in their work. It is extremely important to establish open lines of communication as groundwork for collaboration, and ensure patient advocates are appropriately compensated. Both researchers and patient advocates should establish their expectations when starting to work together. It is important to understand whether the collaboration will be a short- or long-term engagement and both parties should consider time commitments with regard to frequency of meetings as well as the length of meetings.

Ideally, researchers should begin working with patient advocates early in their career^16^. To support these relationships, institutions should build programs to support that connection. A few examples of success include:

- Cornell Community Cancer Partnership: Cornell has developed a program that brings community members affected by, or interested in, cancer together with basic research Ph.D. students^16^. They have monthly seminars where graduate students give presentations in common language or community members describe their experiences living with cancer^17^. The program focuses on science communication and exposing trainees to the human side of cancer.
- Cancer Trainee Advocate Program (CTAP): This program is a resource for how to get trainee programs started at institutions and provides a few examples. The goal is to bring patient advocates from the community together with trainees to have initial discussions about experiences with cancer and their research, respectively.
- *Cellular and Molecular Basis of Disease Course in the Cellular and Molecular Medicine Ph.D. Program at Johns Hopkins University*: During the first year of graduate study, M.D. or Ph.D. faculty members present lectures on human diseases and often bring in a patient to share their perspective. These student–patient interactions are described by students as a highlight of their educational experience.

There are many other examples, both at the national and local level^9^. Once relationships are established, researchers should create a process for maintaining collaboration. Patient advocates enjoy being involved with every step of the research process and including advocates throughout the grant writing process avoids performative advocacy. Researchers should consider patient advocates’ involvement in grants early in the process, not only to support the merit of the grant in the review process, but as a true partner as the grant progresses. This likely involves including them in the budget and/or as a co-author on publications, as appropriate. Having a long-term relationship supports the organic process of patient advocate involvement, which allows time for advocates to become partners in research as the project progresses. A few suggestions for long-standing collaboration are included in Table 2.

**Table 2:** Opportunities for collaboration.

Suggestion	Description
Elevator pitches	Practice elevator pitches, or short 2–3 min talks, with patient advocates. Researchers benefit from working on science communication to explain their research in a more lay-friendly way and patient advocates can ask questions to learn more about the research.
Journal clubs	Create journal clubs where patient advocates and research trainees take turns presenting and learning more about the field. Hearing different perspectives on the same publication will help identify priorities for each group.
Lay abstracts	Write lay abstracts for posters and manuscripts and have a patient advocate help review it. This will not only enhance the relationship between the advocate and researcher but will also make the work more accessible to a larger audience.
Laboratory meetings	Invite patient advocates to laboratory meetings. A patient advocate can ask questions that brings the discussion out of the details and addresses the bigger picture. Before trainees explain their experiment during laboratory meetings, require that they describe how it fits into the broader goal of their project. This will not only help the patient advocate, but it also ensures that the trainees consider and present the rationale for their experiments every time they explain their data. If researchers decide to include patient advocates in laboratory meetings, set the ground rules early by explaining to the patient advocate there will be times when the group needs to delve into details. It may be helpful to ask the patient advocate to write down questions for one on one discussions after the meeting, either with the PI or other members of the laboratory. Considering the patient advocate as an integral laboratory member with their own skill sets and expertise is the most appropriate mindset when including a patient advocate in laboratory meetings.
Teaching	Including a patient advocate as a guest lecture or discussant can support a holistic understanding of tumor biology. Patient advocates can also sit in on mock study sections to support trainees’ explanation of their science to a broader audience, which can help not only for grants that include patient advocates on their panels, but also for study sections that have a broad topic area for funding.

It is important for researchers to consider ways to compensate patient advocates for their time, which may include paying for service, covering travel, or inclusion in a manuscript. Researchers should discuss compensation with patient advocates but understand that there are complexities such as the impacts of receiving disability benefits. The field would benefit from more comprehensive discussions and guidance regarding compensation for patient advocates, including intricacies, appropriate amounts, where funding for advocates comes from.

In the long-term, it would help to have clearer definitions of roles for patient advocates receiving compensation or providing effort in grant applications and research projects. The National Cancer Institute (NCI) should consider explaining how advocacy fits into their NCI Comprehensive Cancer Center designation rubric to more clearly demonstrate the value these relationships bring to cancer centers. There is a potential opportunity to include patient advocates in the context of community outreach and engagement (COE). Laying out expectations of researchers working with patient advocates, as well as the purpose for the interactions, would be a great benefit to the community and improve cancer research overall.

In conclusion, our analysis identified two major barriers to research scientist working with patient advocates. The first is that research scientists do not know how to initiate the relationship and the second is that they are unsure of what the relationship should look like. For cancer researchers, we have highlighted ways to collaborate with patient advocates and outlined examples of established best practices from multiple institutions, a resource that has not been comprehensively outlined before. We hope this document will inspire new relationships and programs as we move towards ensuring the patient voice is considered along the continuum of research. While focused, our discussions were not quantitative in nature. To support implementation of programs, academic centers would likely benefit from a more quantitative description of the approach and outcomes. Future studies should collect detailed demographics and expand the group to diverse demographic groups, especially with respect to gender, age, income, and race. Ultimately, these relationships will improve cancer research and more quickly accomplish our collective goal of improving lives of those who have been diagnosed with cancer. We recommend this relationship would be incorporated as part of the infrastructure of the basic research in cancers.

## Methods

### Virtual workshop

The exercises described herein received ethical approval from Baylor College of Medicine. In June 2021, Theresa’s Research Foundation brought together 25 patient advocates and 10 cancer research scientists for a workshop to discuss the current state of researchers and patient advocate relationships and identify barriers to overcome. Specifically, we focused on defining a “successful” relationship between research scientists and patient advocates. We also discussed examples of successful relationships between research scientists and patient advocates from their personal experiences. Applying lessons learned from these endeavors supports a framework for success. All workshop participants provided written, informed consent to take part in the study and were able to review the manuscript findings prior to submission.

### Conference presentation and manuscript development

We identified key findings from workshop discussions and presented them at the 8th Annual Metastatic Breast Cancer Research Conference in Park City, UT, USA on September 10, 2021, which included about 60 participants in person and over 500 registered virtual attendees. Meeting attendees provided comments and suggestions during the presentation and authors discussed this topic with meeting attendees about throughout the meeting. We combined findings presented at the conference and the discussions we had with meeting attendees to identify solutions to overcome barriers encountered by researchers interested in working with patient advocates.

## Data Availability

The results from the workshops are available from the corresponding author upon reasonable request.
